# Cerebellar tonsil ectopia measurement in type I Chiari malformation patients show poor inter-operator reliability

**DOI:** 10.1186/s12987-018-0118-1

**Published:** 2018-12-17

**Authors:** Braden J. Lawrence, Aintzane Urbizu, Philip A. Allen, Francis Loth, R. Shane Tubbs, Alexander C. Bunck, Jan-Robert Kröger, Brandon G. Rocque, Casey Madura, Jason A. Chen, Mark G. Luciano, Richard G. Ellenbogen, John N. Oshinski, Bermans J. Iskandar, Bryn A. Martin

**Affiliations:** 1 Department of Biological Engineering, University of Idaho, 875 Perimeter Drive MS 0904, Moscow, ID 83844-0904 USA; 2 School of Medicine, University of Washington, Seattle, WA USA; 3 Center for Human Disease Modeling, Duke University Medical Center, Durham, NC USA; 4 Department of Psychology, University of Akron, Akron, OH USA; 5 Department of Mechanical Engineering, University of Akron, Akron, OH USA; 6 Seattle Science Foundation, Seattle, WA USA; 7 Department of Radiology, University Hospital of Cologne, Cologne, Germany; 8 Department of Neurosurgery, University of Alabama at Birmingham, Alabama, USA; 9 Department of Neurosurgery, Helen DeVos Children’s Hospital, Grand Rapids, MI USA; 10 Semel Institute for Neuroscience and Human Behavior, University of California, Los Angeles, CA USA; 11 Department of Neurosurgery, Johns Hopkins University, Baltimore, MD USA; 12 Department of Neurological Surgery, University of Washington, Seattle, WA USA; 13 Department of Radiology & Imaging Science and Biomedical Engineering, Emory University, Atlanta, GA USA; 14 Department of Neurological Surgery, University of Wisconsin, Madison, WI USA

**Keywords:** Cerebellar tonsil, Inter-operator reliability, Morphometric, MRI, Syringomyelia, Type 1 Chiari malformation

## Abstract

**Background:**

Type 1 Chiari malformation (CM-I) has been historically defined by cerebellar tonsillar position (TP) greater than 3–5 mm below the foramen magnum (FM). Often, the radiographic findings are highly variable, which may influence the clinical course and patient outcome. In this study, we evaluate the inter-operator reliability (reproducibility) of MRI-based measurement of TP in CM-I patients and healthy controls.

**Methods:**

Thirty-three T2-weighted MRI sets were obtained for 23 CM-I patients (11 symptomatic and 12 asymptomatic) and 10 healthy controls. TP inferior to the FM was measured in the mid-sagittal plane by seven expert operators with reference to McRae’s line. Overall agreement between the operators was quantified by intraclass correlation coefficient (ICC).

**Results:**

The mean and standard deviation of cerebellar TP measurements for asymptomatic (CM-Ia) and symptomatic (CM-Is) patients in mid-sagittal plane was 6.38 ± 2.19 and 9.57 ± 2.63 mm, respectively. TP measurements for healthy controls was 0.48 ± 2.88 mm. The average range of TP measurements for all data sets analyzed was 7.7 mm. Overall operator agreement for TP measurements was relatively high with an ICC of 0.83.

**Conclusion:**

The results demonstrated a large average range (7.7 mm) of measurements among the seven expert operators and support that, if economically feasible, two radiologists should make independent measurements before radiologic diagnosis of CM-I and surgery is contemplated. In the future, an objective diagnostic parameter for CM-I that utilizes automated algorithms and results in smaller inter-operator variation may improve patient selection.

## Background

**Fig. 1:**
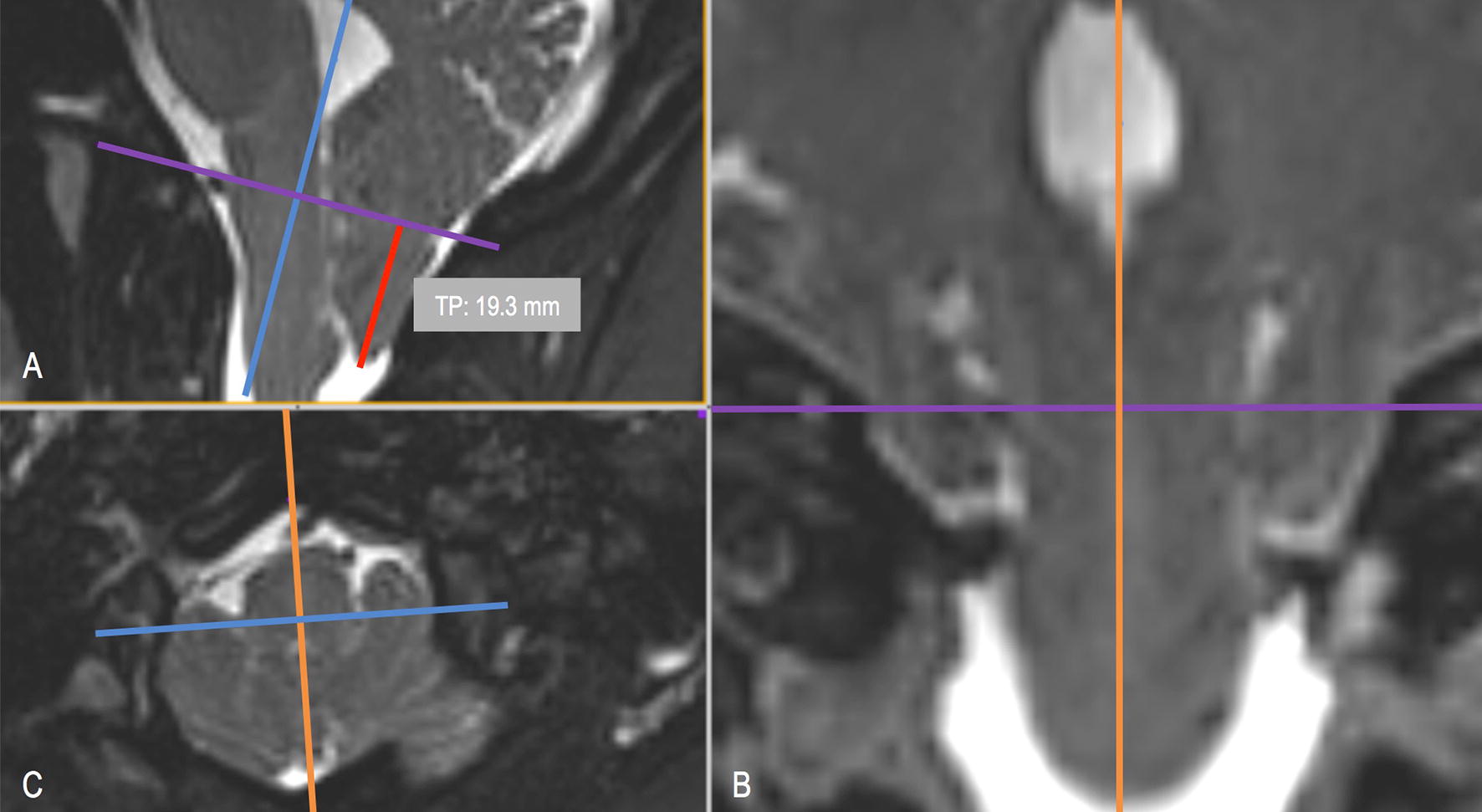
3D multi-planar reconstruction of tonsillar position (TP, red) morphometric measurement for a T2-weighted MRI of subject 27 in this study: **A** mid-sagittal plane with McRae’s line shown as a violet line. **B** Coronal plane with orientation of McRae’s line shown as violet line. **C** Axial plane visualization through McRae’s line showing partial obstruction of cerebrospinal fluid (CSF) space around the spinal cord

Type I Chiari malformation (CM-I) is often defined as caudal descent or herniation of the cerebellar tonsil(s) into the spinal canal > 3–5 mm beyond the basion-opisthion line (McRae’s line) (Fig. 1) [1–9]. Reliability of TP measurements across operators has not been assessed in detail, however, Moore et al. observed reduced variation and higher correlation with TP measurements with reference to the 1st cervical vertebra (C1) arch landmark [10]. Tonsillar position (TP) is regarded as a relatively straightforward measurement for physicians to use in diagnosis or research of CM-I. TP is often measured with reference to McRae’s line in the mid-sagittal plane. It also has been proposed to perform the measurement in the coronal plane, since both cerebellar TPs can be observed together and have been shown sometimes to be asymmetric in CM-I patients [6, 11–13].

The definition of CM-I based on TP has come under scrutiny since several studies employing quantitative measurements have shown that TP does not correlate with the severity of symptoms and the degree of cerebellar TP below the foramen magnum is not heritable in multiplex families with CM-I patients [14–17]. Patients with pronounced TP may present clinically asymptomatic, while patients with minimal or no TP may present with marked symptomatology. With the increased use of MRI, the incidental discovery of asymptomatic patients with TP greater than 5 mm below the foramen magnum is likely to increase [18]. Smith et al. showed that 1, 2, and 3% of adult male, adult female, and pediatric populations, respectively, have TP greater than 5 mm below the FM demonstrating that the cerebellar TP follows a normal distribution and varies significantly by age [6]. In contrast to these cases, there are also patients who present with clinical symptomatology consistent with CM-I without the established radiographic criteria, and consequently, are not considered a CM-I patient by definition. In these cases, the absence of CM-I diagnosis leads to lack of evaluation for surgical treatment that may alleviate symptoms.

Given the importance placed on TP in the diagnosis of CM-I in modern practice, it is crucially important to evaluate TP measurement reliability. The aim of this study was to quantify the inter-operator reliability for the cerebellar tonsillar position among experienced operators, thereby aiding in the decision-making process for physicians and researchers working to diagnose and treat patients with CM-I.

## Materials and methods

### Ethics statement

This study was carried out in accordance with the Declaration of Helsinki (1989) and MRI data acquisition was performed at the University of Wisconsin (UW), the Cleveland Clinic Foundation (CCF), and Emory University (EU). The study was approved by the institutional review board of each institution (IRB #20130226). Prior to scanning, written informed consent was obtained from all subjects. All MRI data were anonymized before being transferred to operators for analysis.

### In vivo MR image acquisition and post-processing morphometric quantification

Axial T2-weighted MR images were obtained without contrast on a 1.5 or 3.0 T scanner with isotropic resolution ranging from 0.750 to 0.875 mm. Image field of view covered the entire craniovertebral junction, upper cervical spine and cerebellum. In total, 33 data sets were used and included 10 healthy controls, with no known neurological or cardiovascular disorders or trauma, and 23 CM-I patients. 12 of the CM-I patients were considered asymptomatic (CM-Ia). 11 CM-I patients were considered symptomatic (CM-Is). The neurosurgeon (MGL or BJI) made a “gold standard judgment” to classify patients as symptomatic based on the presence of sufficiently severe neurological symptoms to warrant corrective cranial/cervical decompression surgery [19]. Patients were classified as asymptomatic that had only mild symptoms or symptoms that were not attributable to tonsillar ectopia and were not recommended for decompression surgery. Although the patients in this group can have symptoms, they are commonly referred to by neurosurgeons as asymptomatic. Patients were excluded that had secondary causes of tonsillar ectopia, such as hydrocephalus, had undergone previous decompression surgery or had implanted cerebrospinal fluid (CSF) shunts.

The 33 MRI data sets were provided to a group of seven expert operators with previous experience (neurosurgery, neurology, and radiology) and previous training in TP measurement. Operators were blinded to the subject status. All operators in the study were male with an average age of 34 years old and with an average of 4.5 years since completing an MD or PhD degree(s). Measurement order for the 33 data sets was randomized for each operator. TP position was measured with respect to McRae’s line using each operator’s preferred software to maintain familiarity and intra-operator consistency. Six different software packages were used to perform TP measurements: Siemens Syngo (Siemens Healthcare GmbH, Erlangen, Germany), McKesson PACS-Horizon Imaging (McKesson, San Francisco, CA, USA), Philips ViewForum (Philips, Amsterdam, Netherlands), RadiAnt (RadiAnt, Poznan, Poland), OsiriX (Pixmeo, Geneva, Switzerland), GE Centricity PACS (General Electric Corp, Boston, USA) and ImageJ [20]. A 3D stack of images was supplied to each operator. For each MRI TP measurement, each operator followed a set procedure: re-format the 3D axial image volume in the sagittal plane, select the mid-sagittal image slice with the maximum caudal extent of tonsillar position and mark the foramen magnum by drawing a line from the basion to the opisthion to most consistently approximate McRae’s line. Craniocaudal distance for each patient was measured to the point of maximal decent perpendicular to McRae’s line. Negative values were reported when TP was measured superior to McRae’s line, and positive values were reported when TP was located inferior to McRae’s line.

### Statistical analysis

Descriptive statistics of TP measurement for 33 MRI data sets for the seven operators were calculated for healthy controls, asymptomatic CM-I patients (CM-Ia), and symptomatic CM-I patients (CM-Is). Intraclass correlation (ICC) was assessed by estimating the level of inter-operator agreement for each MR image using a two-way, mixed model (three levels of patients and two levels of symptom type) [21]. We used patient number as a random effect and operator number as a fixed effect to calculate intra-class correlation (ICC) with a 95% confidence interval (CI) [21, 22]. The ICC was calculated to include all seven operators, as reported. We also performed an additional calculation by including only two operators to verify operator reliability as the number of operators increased. Data were further analyzed for a false negative of CM-I patients in which at least one operator reported a TP value of < 3 mm below the FM, when other operators clearly measured > 5 mm TP below McRae’s line. False positives for the control groups was defined when at least one operator measured TP being > 5 mm below McRae’s line when other operators measured < 3 mm. All statistical analyses were performed using Microsoft Excel for Mac (Version 2016, Seattle, WA) and R (Version 3.4.0, Vienna, Austria).

## Results

**Fig. 2:**
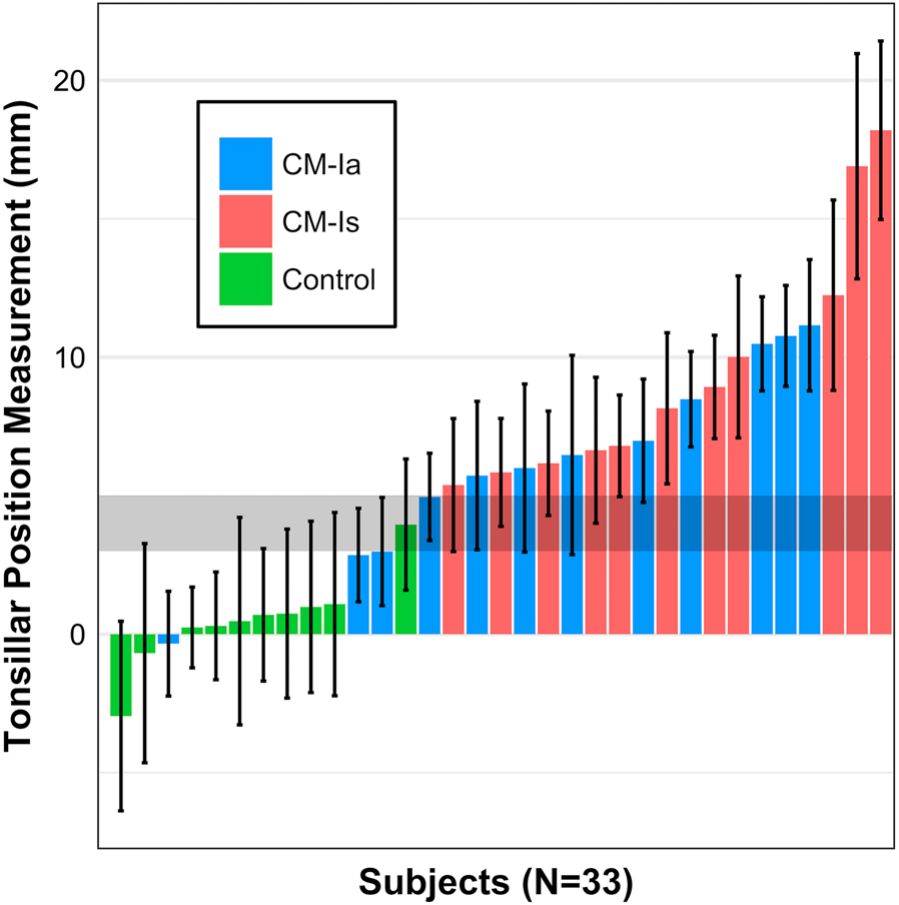
Mean ± 1SD of N = 33 tonsillar position measurements for the seven operators (green = control, red = asymptomatic CM-I patient, blue = symptomatic CM-I patient). Shaded horizontal rectangle indicates TP 3–5 mm

Descriptive statistics for 33 sagittal MRI TP measurements (mm) as measured by seven expert operators are listed in Tables 1 and 2. Table 1 reports the result of seven operator’s measurements for all 33 subjects, Table 2 descriptively compares the overall measurements across seven operators and by subject group (CM-Ia, CM-Is, and control). TP mean ±  standard deviation (SD) for all individuals was 5.66 ± 2.54 mm (Fig. 2). Mean of the maximum and minimum TP measurements for all subjects was 9.17 and 1.43 mm, respectively. The mean ± SD of cerebellar TP measurements for CM-Ia and CM-Is patients in mid-sagittal plane was 6.38 ± 2.19 and 9.57 ± 2.63 mm, respectively. Mean ± SD of cerebellar TP measurements for controls was 0.48 ± 2.88 mm. ICC obtained for all seven operators was 0.83 (95% CI 0.74–0.90, p < 0.001). When only the two most experienced operators were considered in the analysis, the ICC was 0.84 (95% CI 0.70–0.92, p < 0.001). ICC values over 0.75 are considered to have excellent clinical significance [23].

**Table 1:** Descriptive statistics for 33 sagittal MRI tonsillar position measurements (mm) as measured by seven expert operators

MRI subject	Group	Descriptive statistics				
		Range (mm)	Mean (mm)	SD (mm)	Median	IQR
1	CM-Ia	5.80	8.49	1.72	8.30	2.30
2	Control	6.10	0.30	1.95	0.00	3.40
3	CM-Ia	6.00	− 0.34	1.89	0.00	3.10
4	CM-Ia	5.00	2.86	1.69	3.00	3.40
5	CM-Ia	4.80	10.49	1.70	11.10	2.90
6	CM-Is	9.00	10.01	2.92	11.00	1.70
7	CM-Ia	8.70	5.73	2.68	6.00	4.80
8	CM-Ia	7.80	11.16	2.37	11.30	3.40
9	CM-Ia	11.00	6.47	3.60	6.00	6.10
10	CM-Ia	5.00	2.99	1.95	3.00	4.60
11	CM-Ia	7.20	6.99	2.23	7.10	3.60
12	CM-Is	7.60	5.39	2.40	6.00	3.30
13	CM-Ia	5.00	10.77	1.82	11.20	4.10
14	CM-Ia	4.40	4.96	1.58	6.00	2.90
15	Control	7.30	− 2.96	3.42	0.00	7.00
16	CM-Ia	9.20	6.00	3.04	5.00	5.70
17	Control	7.30	0.70	2.39	0.00	4.80
18	Control	5.30	0.24	1.46	0.00	1.00
19	Control	10.00	1.09	3.31	0.00	6.00
20	Control	13.80	− 0.69	3.96	0.00	1.00
21	Control	12.40	0.47	3.75	0.00	6.00
22	Control	6.40	3.96	2.37	2.90	4.70
23	CM-Is	6.10	6.80	1.84	7.00	2.60
24	Control	10.80	0.74	3.05	0.00	3.00
25	CM-Is	7.90	6.64	2.64	7.90	4.00
26	CM-Is	5.20	6.17	1.89	6.00	3.80
27	CM-Is	10.00	18.20	3.22	17.00	5.00
28	CM-Is	9.30	16.90	4.07	14.00	9.00
29	CM-Is	4.40	8.93	1.87	10.00	3.80
30	CM-Is	8.70	8.16	2.73	7.00	4.00
31	CM-Is	6.90	5.84	1.95	5.60	1.40
32	CM-Is	11.00	12.24	3.44	13.00	5.30
33	Control	10.00	0.99	3.09	0.00	4.90

*CM-Ia* type I Chiari malformation asymptomatic, *CM-Is* type 1 Chiari malformation symptomatic, *IQR* interquartile range, *SD* standard deviation

**Table 2:** Descriptive statistics calculated for seven expert operators by MRI subject group across 33 CM-I patients

Measure	Subject group	Operator 1	Operator 2	Operator 3	Operator 4	Operator 5	Operator 6	Operator 7
Range (mm)	All	13.00	28.00	28.00	22.30	23.00	17.00	30.10
	CM-Ia	8.50	10.00	16.20	13.40	10.00	12.00	15.30
	CM-Is	11.80	16.00	14.90	16.70	15.00	13.00	19.90
	Control	2.50	9.00	10.20	6.70	7.00	7.00	13.40
Mean (mm)	All	3.43	4.91	5.87	6.18	8.73	6.55	3.90
	CM-Ia	3.49	6.00	7.55	6.73	8.50	7.67	4.85
	CM-Is	6.10	9.09	10.65	10.37	12.73	8.73	9.34
	Control	0.42	− 0.90	− 1.23	0.93	4.60	2.80	− 3.23
SD (mm)	All	3.65	5.33	6.23	5.48	4.99	4.27	6.90
	CM-Ia	2.96	2.95	4.23	4.15	3.28	3.82	4.43
	CM-Is	3.78	4.40	4.08	4.99	5.28	4.09	5.74
	Control	0.86	2.43	2.78	2.00	2.01	1.89	3.63
Median (mm)	All	2.50	5.50	6.70	6.10	8.00	6.00	3.20
	CM-Ia	3.50	6.00	7.70	6.65	10.00	7.50	4.20
	CM-Is	5.00	8.00	10.50	8.30	10.00	7.00	7.90
	Control	0.00	0.00	− 0.70	0.00	5.50	3.00	− 2.70
IQR (mm)	All	5.30	8.00	10.50	9.15	5.50	7.50	10.55
	CM-Ia	5.83	5.00	6.40	6.95	5.75	6.75	6.90
	CM-Is	5.80	4.00	6.70	7.50	9.00	7.00	8.80
	Control	0.43	2.50	3.18	1.15	3.00	3.00	5.20

*CM-Ia* type I Chiari malformation asymptomatic, *CM-Is* type 1 Chiari malformation symptomatic, *IQR* interquartile range, *SD* standard deviation

**Fig. 3:**
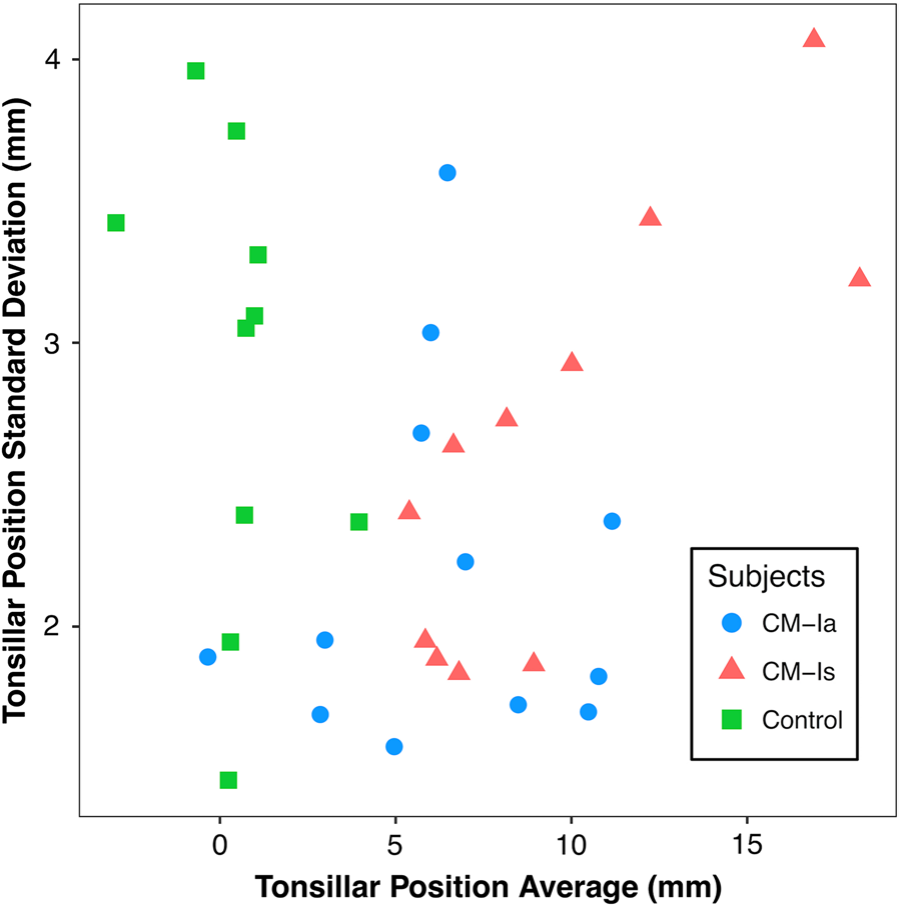
Relation of average tonsillar descent measurement and standard deviation of the measurement for the seven operators. Standard deviation shows no statistical correlation with increase in tonsillar position (R^2^ = 0.004)

**Fig. 4:**
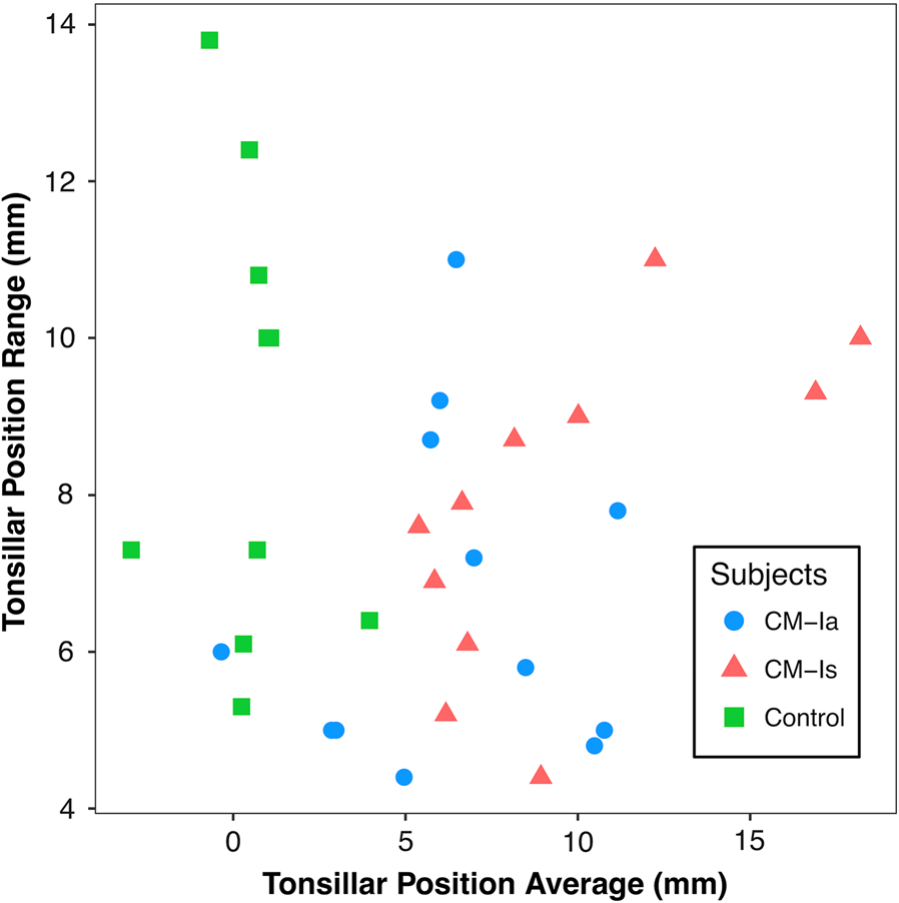
Relation of average tonsillar descent measurement and maximum difference in tonsillar position measurement between the seven operators. Difference in measurements shows slight increase as tonsillar position increases

The average range of TP measurements for all data sets analyzed was 7.7 mm. TP SD and range did not show a statistically significant trend with increasing mean TP measurement obtained for the seven operators (Figs. 3, 4). The range of mean TP ranges (grand range) decreased when the average of TP was increased: for subjects with < 5 mm TP, the grand range was 9.40 mm, however, for subjects with TP > 5 mm, the grand range was 6.60 mm. Likewise, the range of standard deviations also decreased with increasing TP. For TP < 5 mm, the range of standard deviations was 2.50 mm, while the range of standard deviations for TP > 5 mm decreased to 2.37 mm.

In this study, false negatives for CM-I diagnosis were documented when at least one operator reported a TP value of less than 3 mm tonsillar herniation, when other operators clearly measured > 5 mm herniation below McRae’s line. By this definition, eight of the CM-I patients had false negative reports for eight out of 133 measurements. In other words, 6.0% of CM-I patients could have been considered “healthy” by at least one diagnostician when other operators would have radiographically categorized the individual with CM-I. Conversely, healthy controls, when the TP measurement was analyzed where at least one operator measured the TP as being > 5 mm below the FM in the control MRI studies, 8 out of 70 measurements (11.4%) were considered to meet the diagnostic criteria for CM-I (false positive). When the same analysis was repeated with at least two operators making a questionable measurement relative to the group, the number of questionable diagnoses was reduced to 1.5% false negatives for CM-I patients and 4.3% false positives for healthy controls.

## Discussion

The current standard for the radiologic diagnosis of CM-I is based on a static MRI measurement of tonsillar descent 3–5 mm below the McRae line. This study quantifies the variability between seven operators measuring TP for a group of CM-I patients and controls with varying degree of TP relative to the foramen magnum. Overall operator agreement for TP measurements, as measured by ICC, were relatively high, yet there was still a high TP measurement range among expert operators (average TP measurement range of 7.7 mm). These results support that multiple operators should confirm a measurement before arriving at conclusive radiographic CM-I diagnosis.

### Importance of reliability assessment

In the past, research in neuroradiology, neurology, and neurosurgery has examined the inter-rater reliability of operators making MRI measures [11, 24]. Inter-rater reliability allows researchers to quantify to the agreement of measurements made between two or more operators. In this study, the degree of precision for seven operators measuring tonsillar position were collated and analyzed. Many scenarios in the healthcare industry rely on multiple people to collect research or clinical laboratory data, thus it is critical for technologists to have a high degree of consistency when evaluating MR images. The question of consistency, or agreement among the individuals collecting data immediately arises due to the variability among human observers. However, for the TP measurement, there has been little evaluation of the error potential inherent in this diagnostic and its subsequent clinical impact in terms of accurate diagnoses. Moore’s robust assessment of TP measurements with respect to FM, C1, and C2 help to establish a better morphometric measurement for reducing inter-operator variation [10]. There are a number of statistics that have been used to measure intra-rater reliability. The results of this study were measured using the ICC two-way mixed model to account for random patients and fixed operators.

### Significance of results on Chiari diagnosis

Our results show that just one standard deviation for TP measurements across CM-Ia, CM-Is, and controls is approximately ± 2–3 mm. It is possible that this may be a consequence of lack of attention in cases where TP is not extreme, or simply because a smaller mean TP measurement require a smaller standard deviation to be meaningfully accurate. This is important since a borderline CM-I case with 3 mm TP may easily tilt the diagnosis for an individual to be considered for treatment. The false negative rate for operators in this study was approximately 6.0%. The American Association of Neurological Surgeons estimated that approximately 11,000 CM-I patients received surgical treatment in 2007 and 20–40% of these surgeries do not resolve symptoms [25–28]. Conservatively estimating that 50% of patients being evaluated for Chiari undergo surgery implies there are more than 22,000 patients evaluated for Chiari each year. Applying a 6.0% false negative rate to this number yields a potential 1320 missed cases each year that are near the cutoff TP measurement and may not be referred for a detailed clinical assessment of CM-I symptoms.

### Sources of TP measurement error

**Fig. 5:**
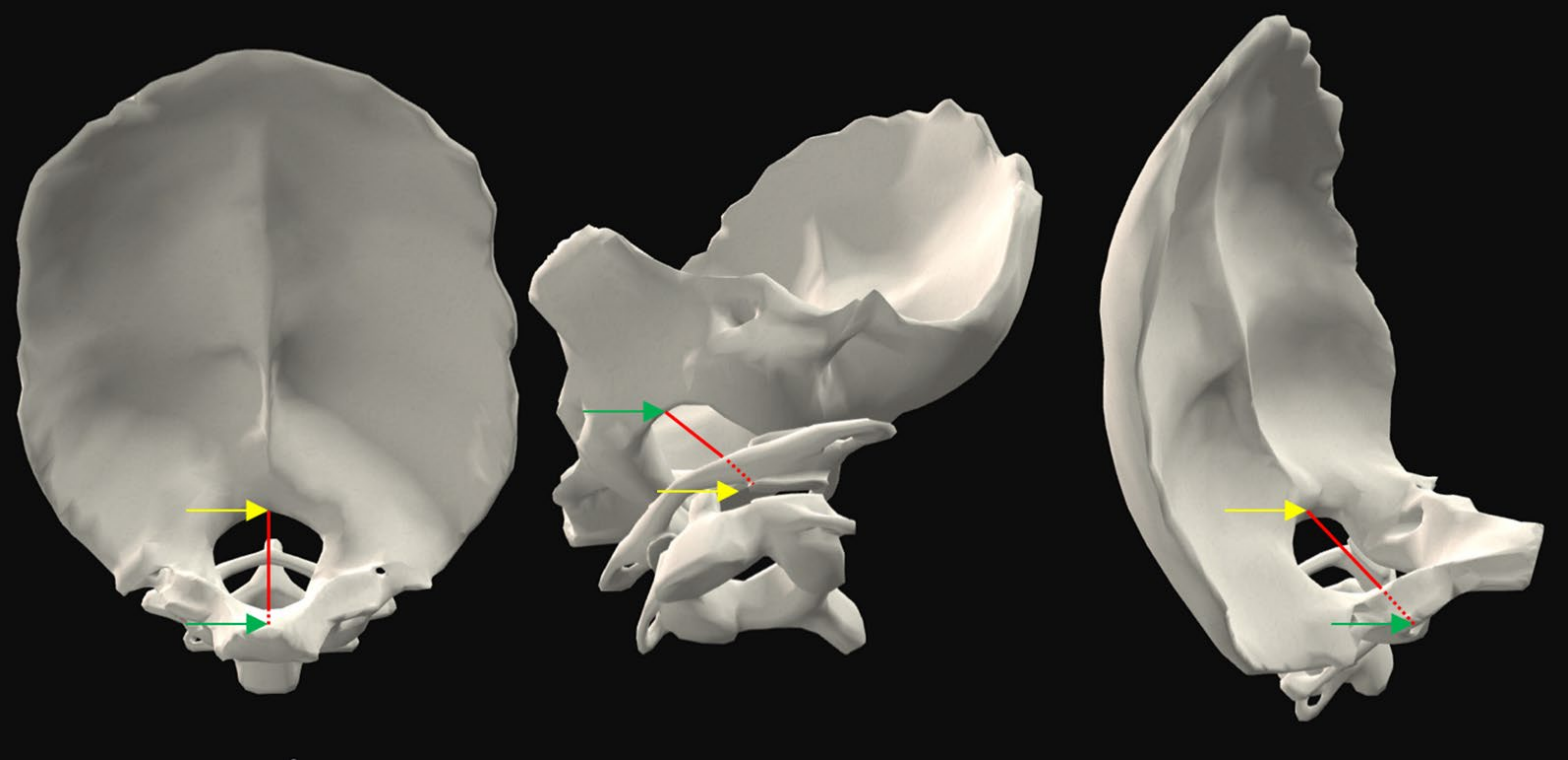
3D reconstruction of posterior cranial fossa and surrounding bony anatomy depicting McRae’s line (red line) drawn between the basion (green arrow) and opisthion (yellow arrow). Suboccipital region of the intracranial space (left), cervical-medullary junction (center and right) (images with permission from BioDigital Human Visualization Platform 1.1)
- Natural variation of TP due to neck orientation, posture and other factors [29].

Morphometric quantification of TP has multiple potential sources of error and difficulty that include:

- MR image type (T1 or T2-weighted) and specific imaging settings such as resolution and slice spacing implemented across scanner system types.
- Selection of mid-sagittal slice and software capabilities for multi-planer reconstruction and placement of the mid-sagittal slice.
- Measurement technique applied to select the basion and opisthion that define McRae’s line.
- Difficulty to determine the grayscale threshold cutoff to define the anatomic landmarks including the cerebellar tonsil tip.
- Complex 3D FM bony morphology that impacts relative TP position with respect to the McRae’s line (Fig. 5).

To ensure operator consistency and help control for additional sources of error, high-resolution T2-weighted images were used in this study only. Approximately half of the operators indicated that they prefer T2-weighted images and half of the operators preferred T1-weighted images for making TP measurements. At present, there is no standard image type that is recommended for TP measurement. Image type may have an impact on TP reliability and should be investigated. To locate the mid-sagittal slice, a set of 3D high-resolution MR images is needed along with software capability for multi-planar reconstruction to correctly select optimal mid-sagittal slice location (Fig. 1). In many clinical settings, a high-resolution 3D MRI may not be possible to obtain, as it requires a longer scanning time. Thus, clinicians often make TP measurement based on imaging with larger slice thickness and with a mid-sagittal slice that is placed by the radiology technician. Due to these factors, we expect that typical TP measurements in the clinical workflow likely have a lower degree of reliability that that found in our study. FM also contributes an additional layer of difficulty to McRae’s line placement between the basion and opisthion. The bony structure at the basion and opisthion has an upward-shaped arch (Fig. 5). Due to the arch-shaped structure, mid-sagittal slice selection will directly impact the location of the basion and opisthion and thereby impact the TP relative to McRae’s line. Note also, invasive surgical treatment further complicates TP measurement by removing part of the reference frame for recreating McRae’s line (opisthion is removed in decompression). The surgical outcome can be difficult to correlate with TP measurement change because this feature is removed.

### Need for an improved Chiari diagnostic

An increasing number of asymptomatic, minimally symptomatic, and doubtfully symptomatic patients are being diagnosed [30–32]. Widespread use of MRI has shown that 1–4% of the US population has radiographic indication of CM-I [6, 33, 34]. However, less than 1 in 30 of these people are regarded as CM-I patients [35]. If left untreated, CM-I can result in CNS damage, especially if the patient develops syringomyelia and resultant neurological deficits. The leading CM-I treatment is a highly invasive brain surgery with a lack of consensus for the “best practice” in surgical techniques [32, 36]. A principal concern within the medical community is that of patients receiving unnecessary operations, specifically if a patient does not meet the diagnostic criteria for the diagnosis. It is imperative that diagnostic criteria for CM-I are further refined to a point of minimizing unnecessary operations that not only have the potential to harm patients but also perpetuate the encouragement of inflated healthcare spending. In addition to the hundreds of millions of dollars in yearly health care costs attributed to CM-I, physical setbacks result in suffering and loss of economic productivity [36–38].

### Impact of false negative and false positive radiographic diagnosis of Chiari

Anecdotally, some patients report being told that a herniation of 3–4 mm is not large enough to cause symptoms and are not referred for further Chiari evaluation (Conquer Chiari correspondence). Delays in diagnosis are further supported by data from the Conquer Chiari Patient Registry, which showed the average time to diagnosis for 768 patients was 3.4 years. The overall effect of delays in diagnosis on eventual patient outcomes is unclear, however it is likely that some patients are encountering significant roadblocks in the diagnostic process due, at least in part, to a lack of awareness of the potential error in measuring TP via MR imaging. The average false positive rate for the operators in this study was 11.4%; however, the rate of false positives (6.0% vs. 1.5%) and false negatives (11.4% vs. 4.3%) drop considerably by simply having one more individual measure the same MR image. Clinically, the impact of false positives is mitigated by subsequent opinion and diagnostic tests for the prevention of surgery. In the meantime, this over-diagnosis results in patient anxiety and subsequent testing that results in additional costs, risks, and anxiety. Ultimately, the TP measurement will be only one factor in a more thorough consideration of decompression surgery.

### Diagnostic problem of asymptomatic Chiari

Based on comprehensive review of the literature, at least 50% of patients that come to medical doctors for CM-I assessment are considered asymptomatic [10, 12, 14, 17, 19, 24, 30, 33, 39, 40]. Increasing use of MRI has revealed that large tonsillar descent can be accompanied by no symptoms and vice versa [2, 15, 41]. Speer et al. estimated the prevalence of CM-I to be 200,000 persons, a value less than 0.06% of the United States population, but as already mentioned, more recent studies indicate that upwards of 1–4% of the population may meet the diagnostic criteria for anatomic CM-I [6, 34, 42, 43]. More specifically, recent radiological findings indicate that 1% of adult male, 2% of adult female, and 3% of pediatric population meet the radiographic diagnosis of CM-I [33]. Thus, CM-I prevalence is upwards of two orders of magnitude less than the radiological findings of tonsillar descent [19]. It is clear that additional diagnostic measures or diagnostic methods such as machine learning are needed to accurately detect CM-I and thereby aid in the decision of treatment options [40, 44]. In addition to static morphometric analysis, there is a need for dynamic MRI-based methods that identify symptomatic CM-I and correspond to severity of symptoms. Some proposed measurement methods include phase-contrast MR imaging to quantify CSF velocities [9, 45–51], time-slip or STAMP MRI methods [52], and cardiac-related neural tissue motion [53–57].

## Limitations

The principle limitation in our work is that each operator used different software packages to perform the measurements. Also, the trained expert operators had varying degree of experience with radiographic measurement of TP. Differences in operator training could contribute to variation in TP measurement. Furthermore, only seven operators made the measurements. It would be beneficial to check consistency of results across a greater number of operators and for various operator cohorts with identical previous training and experience. We did not check measurement consistency between software programs since it is expected that different software is used in daily practice across clinical centers and operators. The accuracy and resolution of software could be different and influence the measurement result. Our aim was not to analyze the reliability of individual software packages. Our primary goal was to capture a snapshot of clinically applied measurements as one may experience in day-to-day activities. We feel that it is reasonable to assume natural variation in training level and software used for the diagnosis and treatment of CM-I across centers.

## Conclusion

These results demonstrate a high degree of variability in TP measurement among expert operators and support that, if economically feasible, two radiologists should make independent measurements before the radiologic diagnosis of CM-I and surgery is contemplated. An objective diagnostic parameter is needed for use with traditional diagnostic methods that more clearly predicts diagnosis and symptomatology, as well as reducing inter-operator variation across imaging centers.

## Abbreviations

C1: 1st cervical vertebra
CI: confidence interval
CM-Ia: asymptomatic Chiari malformation type I
CM-Is: symptomatic Chiari malformation type I
CM-I: Chiari malformation type I
CSF: cerebrospinal fluid
FM: foramen magnum
ICC: intraclass correlation coefficient
IQR: interquartile range
SD: standard deviation
TP: tonsillar position
